# Increasing of *Q* Factor of Quasi‐Bound States in the Continuum With Increased Asymmetry Parameter in a Silicon Metasurface Array

**DOI:** 10.1002/nap2.70208

**Published:** 2026-07-15

**Authors:** Yi Tian, Qin Fu, Shuai Feng, Qingguo Du, Zhengying Li, Fei Xu

**Affiliations:** ^1^ School of Information Engineering Wuhan University of Technology Wuhan China; ^2^ School of Science Minzu University of China Beijing China; ^3^ National Engineering Research Center of Optical Fiber Sensing Technology and Networks Wuhan University of Technology Wuhan China; ^4^ College of Engineering and Applied Sciences Nanjing University Nanjing China; ^5^ National Laboratory of Solid State Microstructures Nanjing University Nanjing China

**Keywords:** metasurface, quality factor, quasi‐bound states in the continuum

## Abstract

Quasi‐bound states in the continuum (qBICs) can support resonances with high quality (Q) factor. However, the Q factor of qBICs decreases rapidly as the asymmetry parameter α increases. Traditionally, maintaining a high Q factor requires a small α, which brings significant challenges for nanofabrication. In contrast, our study finds an increase in the Q factor with increasing of α in a metasurface array composed of Si blocks with circular notch. For small asymmetry parameter (α≤0.14), the Q factor of the qBIC follows the inverse square law. However, when α>0.14, the Q factor increases gradually. Through detailed analysis of the coupling strength and electromagnetic field distributions, we attribute the increase of the Q factor to the coupling between the qBIC and the lattice surface mode (LSM), which arises from the alignment between the diffraction wavevector and the reciprocal lattice vector. In this case, the Q factor under large α can reach nearly $10^{5}$ and is maintained over a wide range of α. Our work offers a new pathway for the design of high Q qBIC resonances at large asymmetry and contributes to other research field related to the high‐Q nature of qBIC in photonics.

## Graphical abstract

Quasi‐bound states in the continuum (qBICs) can support resonances with high quality (Q) factor. Traditionally, maintaining a high Q factor requires a small α, which brings significant challenges for nanofabrication. In contrast, our study finds an increase in the Q factor with increasing of α in a metasurface array composed of Si blocks with circular notch.

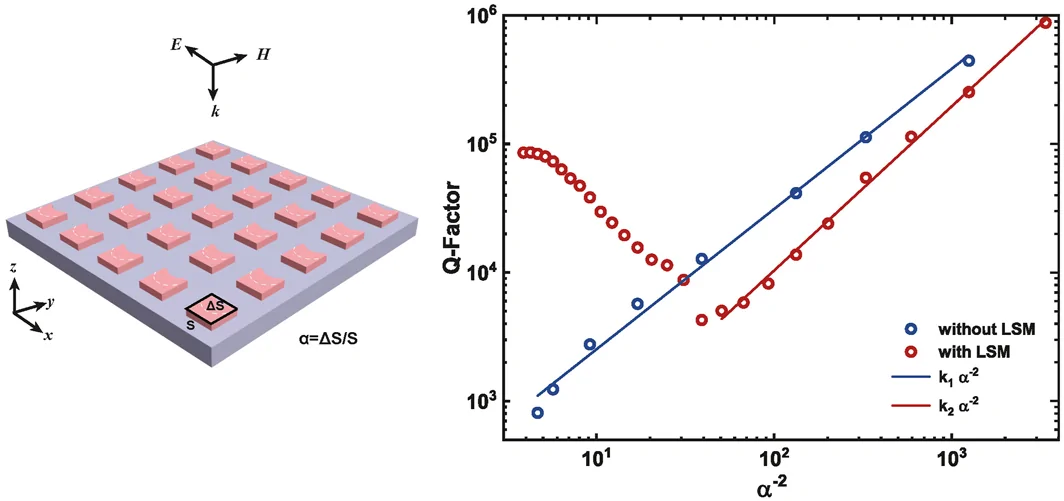

## 1 Introduction

The bound states in the continuum (BICs) originated from the energy eigenvalue problem of a particle in a spherical quantum well are universal physical phenomena, existing within the radiation continuum while remaining completely decoupled from it, resulting in an infinitely large Q factor [1, 2]. This unique property has led to a wide range of researches and applications in the fields of acoustics, electronics, and nanophotonics [3, 4, 5, 6, 7]. A perfect BIC is not detectable, but it can be disrupted by introducing defects, converting it into a leaky mode known as quasi‐bound states in the continuum (qBICs), which is characterized by an extremely high Q factor [8]. This conversion is critical for utilizing BICs, and the approach to achieving it varies depending on the specific type of BICs. In nanophotonics, a prominent kind of BIC is symmetry‐protected BIC (SP‐BIC), which only occurs at Γ points in momentum space [9]. This mode cannot couple to free space due to the symmetry mismatch between the mode's spatial profile and the outgoing plane waves [10]. To convert a SP‐BIC into a qBIC, one can either slightly break the symmetry of the excitation field through oblique incidences [11, 12] or modify the spatial symmetry of the structure, which will enable the coupling of the SP‐BIC to free space and convert it into a qBIC. [13, 14, 15, 16]. Another kind of BIC is accidental BIC, which can appear anywhere in the momentum space. This kind of BIC originated from accidental destructive interference between different modes [2]. Accidental BICs can be manipulated or transformed into qBICs by adjusting system parameters [17, 18, 19].

Achieving high‐Q qBIC resonances has been studied intensively. *Koshelev* et al. rigorously established a direct connection between BICs and Fano resonances, providing an analytical explanation for the inverse square relationship between the Q factor and the asymmetric parameter [20]. High Q‐factor could be obtained for small asymmetric parameter. *Bogdanov* et al. demonstrated that BICs can also be generated when destructive interference occurs between two modes that are strongly coupled to each other, indicating that high‐Q qBIC resonances can be achieved under strong coupling conditions [21]. *Han* et al. discovered that, at larger asymmetry, multiple new states of extended BICs emerge beyond the typical inverse‐square law where high Q‐factor could also be obtained [22]. *Huang* et al. demonstrated that introducing perturbations in the zero‐eigenfield region of SP‐BICs can realize ultrahigh‐Q qBICs [23]. *Kühne* et al. investigated the impact of nanofabrication tolerances on BIC metasurfaces, revealing how design choices influence the robustness of high‐Q resonances [24]. *You* et al. showed that different symmetry‐breaking strategies in all‐dielectric metasurfaces allow tuning of Q‐factor scaling laws, whereas *Kutuzova* and *Rybin* explored how multipolar contributions affect the Q‐factor dependence on asymmetry, expanding the conventional understanding of the scaling relationship [25, 26].

Metasurfaces, consisting of artificially designed periodic subwavelength structures, facilitate efficient wavefront control and resonant transmission engineering due to their strong interaction with electric and magnetic fields [27, 28, 29]. This enhanced light–matter interaction is typically achieved by resonant effects, which are determined by the design of the unit cells and the lattice period. Consequently, a well‐designed metasurface can support a variety of resonance modes, including surface plasmon resonance [30, 31], surface lattice resonance [32, 33], Mie resonance [34], and anapole modes [35, 36, 37]. To enhance the Q factor of resonances and reduce radiative losses in both metasurfaces, introducing out‐of‐plane resonances has emerged as an effective strategy [38, 39, 40].Recently, there are significant interest in studying BICs in metasurfaces. The high‐Q nature of BICs have found wide‐ranging applications in various fields like lasers [41, 42, 43], sensors [14, 44, 45] and opto‐electronic devices [46]. Several recent works have demonstrated robust high‐Q qBIC resonances through precise geometric tuning [47, 48], manipulation of topological charges [49], energy band modulation [50] or the introduction of additional perturbation layers [51]. Traditionally, a small asymmetry parameter results in weak coupling between the qBIC and free space, leading to a resonance with a high Q factor. However, such a small asymmetry parameter brings extra challenges in fabrication of metasurfaces. Therefore, achieving high‐Q qBIC resonances with large asymmetry parameters becomes an interesting topic among researchers, which could enhance the fabrication tolerance while maintaining the high Q‐factor.

In this study, we found an increase in the Q‐factor of the qBIC with the increase of the asymmetry parameter in the proposed metasurface array. For small asymmetry (α≤0.14), the Q factor follows the traditional inverse‐square law, governed by the coupling strength between ${\text{qBIC}}_{1}$ and free space. When α>0.14, an increase of Q factor is found. A detailed analysis of the coupling strength and electromagnetic field distributions found that the increase in the Q factor results from the coupling between the qBIC and the Lattice Surface Mode (LSM), which arises from the matching of the diffraction wavevector with the reciprocal lattice vector. This anomaly increasing in the Q factor provides a potential way for achieving high‐Q resonances with large symmetry parameters. Moreover, the relatively slow change of the Q factor with regard to the asymmetry parameter enhances the fabrication tolerance for imperfections of the structural symmetry. When applied to refractive index sensing, the bulk sensitivity for metasurace array with a large asymmetry parameter can reach 605 nm/RIU, which is comparable to that of the array with small asymmetry parameter.

## 2 Design of qBIC

The schematic of the proposed silicon metasurface array arranged in square lattice is shown in Figure 1a. X‐polarized light is incident on the structure along ‐z direction. The unit cell of the array is shown in Figure 1b which is composed of a silicon square block placed on a ${\text{SiO}}_{2}$ substrate and the metasurface array is merged in a semi‐infinite film. The structural asymmetry is introduced by a circular notch, with the asymmetry parameter α defined as the ratio of the notch area to the area of the square block (ΔS/S), serving as a measure of the degree of symmetry breaking induced by the notch. The thickness of the silicon block is indicated as t. In order to show the details of the unit cell, top‐down view of the ⋅ unit cell is shown in Figure 1c. The period is indicated as P, the side length of the square block, the radius of the circular notch and the distance between the center of the block and the circle are indicated as a, r, and d, respectively. The refractive index of the ${\text{SiO}}_{2}$ substrate and the silicon block are fixed to 1.45 and 3.5, a, r, and P are fixed at 450, 230 and 900 nm, respectively. The thickness t is set to 30 nm because a low contrast strategy can suppress scattering losses and enhance the overall Q‐factor and performance of practical devices [52]. The refractive index of the semi‐infinite film is set to 1.45, which creates a homogeneous environment that can maximize the diffractive coupling due to the phase matching of the in‐plane diffraction orders in the lower and upper media. All simulations are calculated in COMSOL Multiphysics with Finite Element Method (FEM). The periodical boundary conditions are used in the x and y directions, whereas perfect matching layers (PMLs) are employed in the z direction. The global maximum and minimum mesh sizes are set to 170 and 5 nm, respectively. For the three silicon blocks, a finer mesh is employed, with maximum and minimum mesh sizes of 50 and 5 nm, respectively. A convergence test is conducted to verify the reliability of the mesh, with detailed results provided in Supporting Information S1: S1.

**FIGURE 1:**
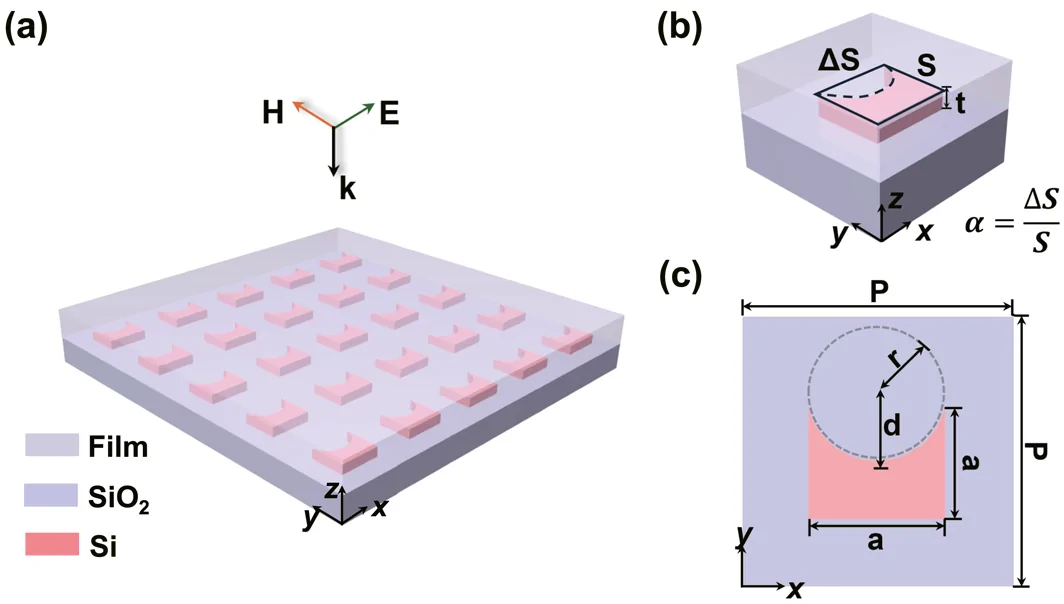
(a) Schematic of the proposed metasurface array illuminated by a x‐polarized plane wave. (b) Schematic of a unit cell of the metasurface, the area of the circular notch and the square is represented in figure as ΔS and S. The thickness of the silicon block is represented as t. The asymmetry parameter α is defined as ΔS/S. (c) Top‐down view of the unit cell. The structural parameters of this schematic are P = 900 nm, r = 230 nm, a = 450 nm, and d = 200 nm.

To investigate the BIC modes within the regulate symmetric metasurface array without circular notch, the band structure is calculated and shown in Figure 2a. Four bands are found with normalized resonance frequencies located near 0.685. ${\text{Mode}}_{1}$ and ${\text{Mode}}_{4}$ are BIC modes at the Γ point. ${\text{Mode}}_{2}$ and ${\text{Mode}}_{3}$ are degenerated modes at the Γ point. The far‐field polarization maps of ${\text{Mode}}_{1}$ and ${\text{Mode}}_{4}$ are displayed in the right panel of Figure 2a which both exhibit distinct vortex pattern with vortex center at Γ point. Clearly, ${\text{Mode}}_{1}$ and ${\text{Mode}}_{4}$ are the typical symmetry protected BICs.

**FIGURE 2:**
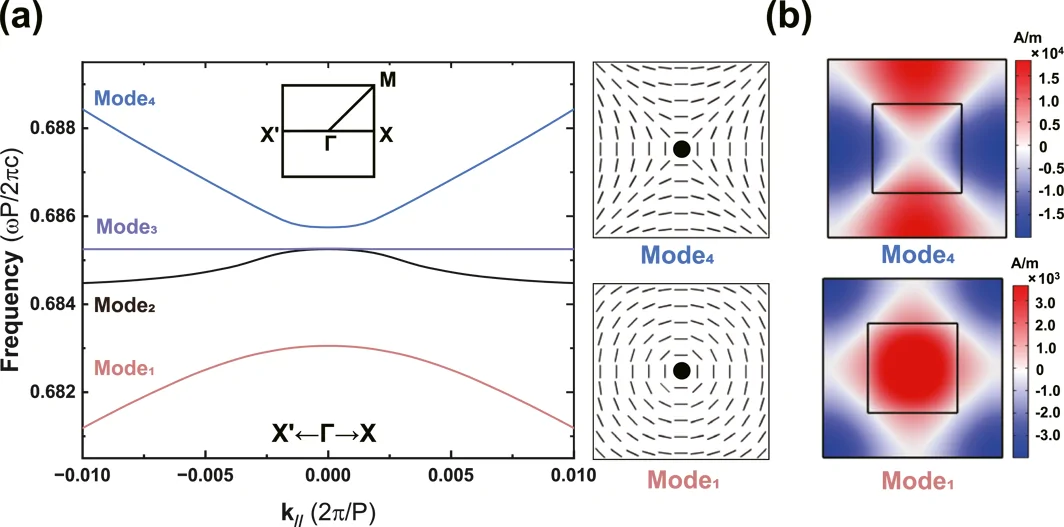
(a) Calculated band structure of the metasurface with $C_{4v}$ lattice in the vicinity of the Γ point in momentum space, where the inset shows the wave vector scanning region of the first Brillouin zone. The right panel shows the far‐field polarization states in the momentum‐space of ${\text{Mode}}_{1}$ and ${\text{Mode}}_{4}$. (b) The eigenmodes profile ($H_{z}$) of ${\text{Mode}}_{1}$ and ${\text{Mode}}_{4}$ on the bottom of the silicon block.

Additionally, the nonradiative nature of these two BICs can be well understood through analyzing the symmetry properties of the mode profile of magnetic filed in z direction $\left(H_{z}\right)$ of each of the bound states. The structure is invariant under symmetry operations from the $C_{4v}$ point group. For a two dimensional lattice, six types of eigenmodes can be supported including two double‐degenerate modes $E_{1}$, $E_{2}$, four nondegenerate modes $A_{1,2}$ and $B_{1,2}$ [13]. A $C_{4v}$ lattice exhibits eight symmetry operations, indicated as $\left\{E,C_{4},C_{4}^{-1},C_{2},\sigma_{x},\sigma_{y},\sigma_{d}^{\prime },\sigma_{d}^{\prime \prime }\right\}$. The outcomes of these operations can be represented as 1 or −1, indicating symmetric overlap and antisymmetric overlap, respectively. Details of these symmetry operations and the character table of each irreducible representation is shown in Supporting Information S1: S2. According to the field profile $\left(H_{z}\right)$ shown in the upper panel of Figure 2b, the symmetry property of ${Mode}_{4}$ can be represented by {1,−1,−1,1,1,1,−1,−1}. This indicates that ${Mode}_{4}$ corresponds to the $B_{1}$ irreducible representation of the $C_{4v}$ point group. Similarly, according to the lower panel of Figure 2b, ${Mode}_{1}$ exhibits a symmetry property represented by{1,1,1,1,1,1,1}, which aligns with the $A_{1}$ irreducible representation. By analyzing these irreducible representations, it is evident that ${Mode}_{1}$ and ${Mode}_{4}$ are decoupled from the radiation continuum. This decoupling arises from their orthogonality to mode profile of the x or y‐polarized plane waves. By introducing a circular notch along the y‐axis, both ${\text{Mode}}_{1}$ and ${\text{Mode}}_{4}$ degenerates into qBIC modes because their symmetry preservation are disrupted, enabling radiative leakage of the ideal BIC modes into free space.

## 3 Results and Discussions

To illustrate the characteristics of qBIC modes with asymmetry parameter α, the silicon metasurface array with d=420 nm (α=0.0283) is chosen as an example. As shown in Figure 3a, the transmission spectrum under normal incident exhibits two dips at 1312.038 nm and 1316.35 nm, as well as a broad dip at 1313.61 nm. The resonances at 1316.35 nm and 1312.038 nm are two resonances from qBIC modes indicated as ${\text{qBIC}}_{1}$ and ${\text{qBIC}}_{2}$, respectively. In our study, the ${\text{qBIC}}_{1}$ resonance is studied in detail, as the extremely narrow linewidth of the ${\text{qBIC}}_{2}$ resonance requires a much finer simulation resolution and thus significantly increases the computational cost. The spatial distributions of electric and magnetic fields for ${\text{qBIC}}_{1}$ and ${\text{qBIC}}_{2}$ on the x‐y plane at the bottom surface of the silicon block and on the x‐z plane at the center of the silicon block are shown in Figure 3b. In each subfigure, the corresponding displacement currents are also shown, with arrows indicating the current directions for clarity. As shown in the first column of Figure 3b, the magnetic field distribution of ${\text{qBIC}}_{2}$ is concentrated along the four unit cell edges. The field distributions show that the current loops around adjacent magnetic charges move in opposite directions, indicating opposite polarizations. This field distribution matches the characteristics of ${\text{Mode}}_{1}$ shown in Figure 2b, indicating that the introduction of the circular notch converts ${\text{Mode}}_{1}$ into the ${\text{qBIC}}_{1}$ resonance. As shown in the second column of Figure 3b, the magnetic fields are concentrated at the center and each corner of the silicon block. This field distribution matches the characteristics of ${\text{Mode}}_{2}$ shown in Figure 2b, indicating that the introduction of circular notch converts the BIC ${\text{Mode}}_{2}$ into the ${\text{qBIC}}_{2}$ resonance. To investigate the mechanism behind the ${\text{qBIC}}_{1}$ resonance, multipole decomposition is performed with detailed formulas provided in Supporting Information S1: S3. As illustrated in Figure 3c, the ${\text{qBIC}}_{1}$ resonance is primarily driven by the electric quadrupole (EQ) component, with a secondary contribution from the magnetic dipole (MD).

**FIGURE 3:**
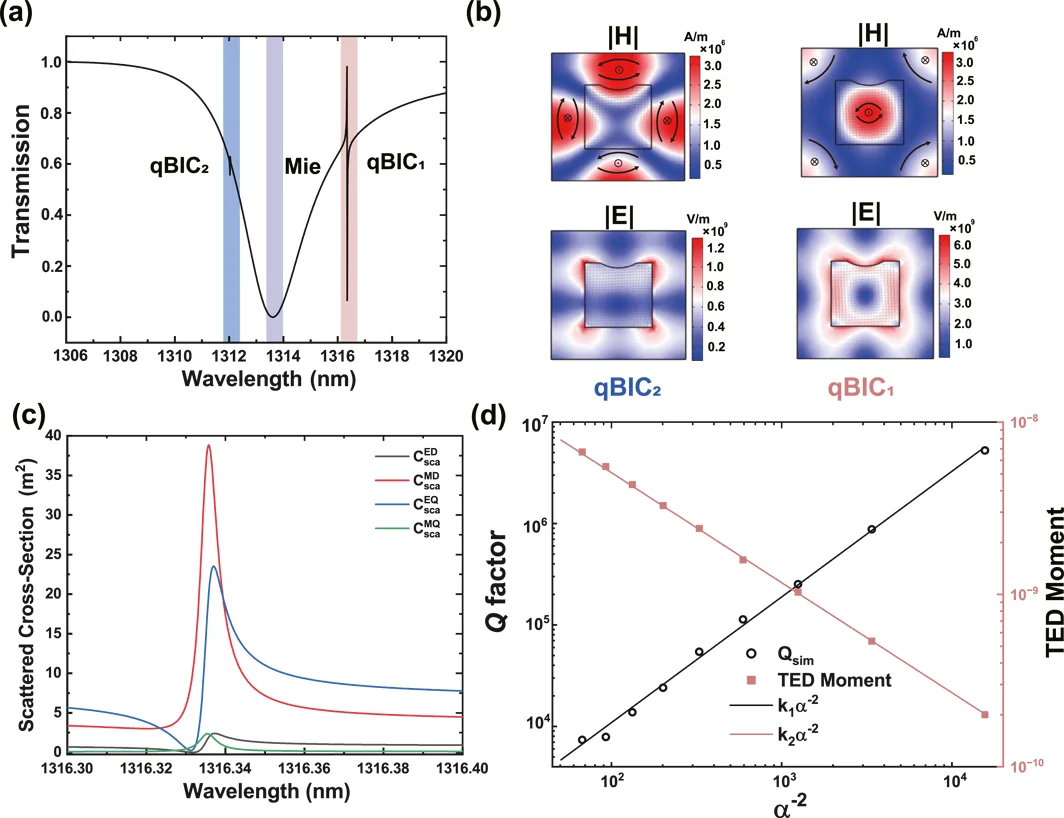
(a) Simulated transmission spectra of the metasurface with a small asymmetry parameter(d = 420 nm, α=0.0283). (b) Corresponding distributions of electric and magnetic fields for these two modes on the bottom of the silicon block, with colors representing field intensities and arrows indicating displacement currents. (c) Normalized multipole decomposition results of ${\text{qBIC}}_{1}$ under Cartesian coordinates. (d) Simulated Q factor and coupling strength vary with $\alpha^{-2}$. The black hollow points and red square points represent the calculated results, whereas the solid line shows the outcome of linear fitting.

To investigate the evolution of ${\text{qBIC}}_{1}$ with regard to the asymmetry parameter α, the changing of Q factor and coupling strength are studied in detail. The Q factor serves as a measurement of the coupling strength of the qBIC and radiation channel. Stronger coupling between qBIC mode and the free space leads to a reduction in the Q factor. Mostly, only small asymmetry parameters are considered because of the high Q nature of the resonance. The Q factor and the coupling strength of ${\text{qBIC}}_{1}$ with α within the range from 0.008 to 0.14 are shown in Figure 3d. The Q factor is derived from the Fano fitting of the transmission spectrum using the equation below [53, 54, 55]:

(1) $$T\left(\lambda \right)=T_{0}+A_{0}\frac{{\left[q+2\left(\lambda -\lambda_{0}\right)/\tau \right]}^{2}}{1+{\left[2\left(\lambda -\lambda_{0}\right)/\tau \right]}^{2}}$$

where λ is the resonant wavelength, τ is the resonance line‐width, $T_{0}$ is the transmission offset, $A_{0}$ is the continuum‐discrete coupling constant, and q is the asymmetry parameter of the resonance. The Q factor is calculated by $\lambda_{0}/\tau$. The coupling strength is used to evaluate of the coupling between ${\text{qBIC}}_{1}$ and the free space which can be described as the overlap between the incident wave field and the eigenstate field. Here, a time dependent electromagnetic field in the form $e^{-i\omega t}$ is used to represent the field of incident plane wave while displacement current density of the bottom surface is used to represent the eigenstate field. Consequently, the coupling strength c could be expressed as [9, 56].

(2) $$c\propto \int_{V}\left[\left[j\left(r\right)\cdot E\right]e^{\mathrm{ikz}}\right]dr$$

where V is the volume of the notched silicon square, j(r) is the displacement current density of the eigenstate field, and $Ee^{\mathrm{ikz}}$ represents a normally incident light propagating along the z‐direction, with the polarization vector E lying in the x‐y plane. In our simulation, the metasurface is illuminated by a x‐polarized light. Thus, the integral in Equation (2) will generate the total electric dipole (TED) moment along x‐axis of the silicon block, which directly quantifies the coupling strength through their proportional relationship. As shown in Figure 3d, the Q factor is proportional to $\alpha^{-2}$, which coincides with that of other reports. With regard to the TED moment, it is inversely proportional to $\alpha^{-2}$. Both Q factor and TED moment have similar relationship with $\alpha^{-2}$, which implies that the inverse square law governing the Q factor actually originated from coupling strength between the incident wave field and the eigenstate field.

The Q factor and TED moment of ${\text{qBIC}}_{1}$ with continuous increasing of α are also calculated and shown in Figure 4a. Surprisingly, the Q factor demonstrates a saturating growth trend with increasing α, deviating from the conventional inverse‐square law. Consequently, the high Q factor with large asymmetry parameter relieves the fabrication difficulties of high‐Q qBIC metasurfaces, where the high Q factor can only be obtained for a structure with a small asymmetry parameter. Simultaneously, this saturation behavior of the Q factor also enhances the tolerance to manufacturing imperfections of the structural symmetry. The effects of small variations in asymmetry parameter on the ${\text{qBIC}}_{1}$ resonance under both large and small asymmetry parameters are investigated and shown in Supporting Information S1: S4. One may notice that there is a slight mismatch at α=0.1407, the TED moment exhibits a corresponding decrease, which implies that the coupling between ${\text{qBIC}}_{1}$ and freespace is getting weaker. The slight mismatch is explained in Supporting Information S1: S5 in detail.

**FIGURE 4:**
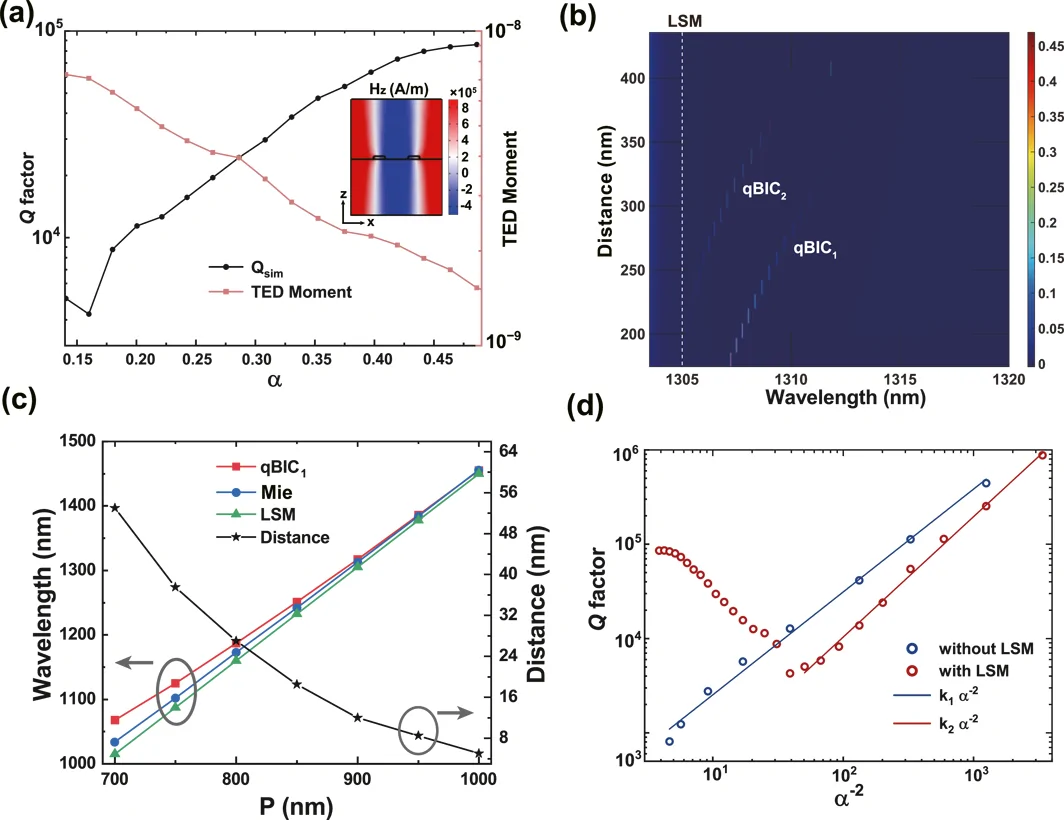
(a) The simulated Q factor and coupling strength vary with α. The right panel shows the electric and magnetic field distributions on the x−z plane of ${\text{qBIC}}_{1}$. (b) Extinction spectrum varies of d with the color indicates extinction. The wavelength of LSM is highlighted in the figure using white dashed line. (c) The wavelengths of ${\text{qBIC}}_{1}$, Mie, and LSM as a function of period P are indicated with red, blue, and green lines, respectively, whereas the distance between the wavelength of ${\text{qBIC}}_{1}$ and LSM is indicated by a black line. (d) The Q factor of ${\text{qBIC}}_{1}$ varies with asymmetry α with (P = 900 nm) and without (P = 700 nm) the influence of LSM.

To investigate the origin of this behavior, α=0.4638 (d=200nm) was selected as an example with electric field shown in the inside panel of Figure 4a. The magnetic field $\left(H_{z}\right)$ along the −z direction appears at the center of the Si blocks. On both sides of it, the magnetic field points in the +z direction. This field distributions indicates that the mode is propagating along the surface in the x‐direction, which confirms that the LSM is involved. The LSM arises from momentum matching between high‐order diffracted light and the periodic lattice, and its in‐plane propagation naturally leads to the observed field distribution outside the structure. As discussed previously, the relationship between the Q‐factor and the asymmetry parameter α can be understood through the coupling strength between the ${\text{qBIC}}_{1}$ resonance and free space. A decrease in this coupling strength implies that the interaction between ${\text{qBIC}}_{1}$ and free space is suppressed due to the energy being redirected into the LSM. To support this point, the extinction spectra for different distances d are calculated and shown in Figure 4b, where the wavelengths of LSM is highlighted by white dashed line (formula to calculate the wavelength of LSM is presented in S6). As the asymmetry parameter increases, distinct extinction peaks gradually appear for both the ${\text{qBIC}}_{1}$ and ${\text{qBIC}}_{2}$ resonances. This clearly demonstrates that the coupling between the qBIC resonances and the LSM makes more energy propagate along the surface, reducing radiation into free space. The LSM provides an additional nonradiative channel for ${\text{qBIC}}_{1}$ [57, 58, 59, 60]. During the approaching, the coupling between ${\text{qBIC}}_{1}$ and LSM is getting stronger, consequently, the coupling between qBIC and free space is getting weaker, resulting in an increase of the Q factor. The mechanisms behind ${\text{qBIC}}_{1}$ and ${\text{qBIC}}_{2}$ are identical, detailed discussions about ${\text{qBIC}}_{2}$ can be found in Supporting Information S1: S7. According to the above analysis, the increase of the Q factor occurs as the ${\text{qBIC}}_{1}$ resonance continuously approaching the wavelength of the LSM when the asymmetry parameter α increases. The inflection point of the increase could be controlled by tuning the wavelength distance between the ${\text{qBIC}}_{1}$ resonance and the LSM (Detailed discussions regarding the influence of period P and thickness t are included in Supporting Information S1: S8). To further verify the contribution of LSM, the wavelength distance can be enlarged to decouple ${\text{qBIC}}_{1}$ and LSM by reducing the period P. As shown in Figure 4c, when the period P increases from 800 to 1000 nm in a step of 50 nm, both the ${\text{qBIC}}_{1}$ and TD resonances redshift. However, because the ${\text{qBIC}}_{1}$ resonance shifts more slowly than the TD and LSM, the wavelength distance between them gradually decreases. When P = 700 nm, the wavelength distance of ${\text{qBIC}}_{1}$ and LSM reaches 53 nm, where the influence of LSM is eliminated. As shown in Figure 4d, without LSM, the Q factor of qBIC follows the inverse square law even at large asymmetry parameter α. This contrasts with the previously discussed case of P = 900 nm, where the Q factor increases at large α. This observation further confirms the participation of the LSM in this phenomenon. Moreover, an additional case with a noncenter‐symmetric notch is presented in Supporting Information S1: S9.

To describe the spectral response, a transmission model based on temporal coupled‐mode theory (TCMT) is first constructed. In this framework, the transmission amplitude is formulated as the coherent sum of a direct transmission background, a broad Mie resonance, and a narrow qBIC resonance [61, 62]:

(3) $$t\left(\omega \right)=c+\frac{B_{M}}{\omega -\overset{˜}{\omega_{M}}}+\frac{B_{Q}}{\omega -\overset{˜}{\omega_{Q}}},T\left(\omega \right)=|t\left(\omega \right){|}^{2}.$$

where *c* is the nonresonant background, $\overset{˜}{\omega_{M}}=\omega_{M}+i\gamma_{M}$ and $\overset{˜}{\omega_{Q}}=\omega_{Q}+i\gamma_{Q}$ represent the complex frequencies of the Mie and qBIC resonance, respectively. $B_{M}$ and $B_{Q}$ describe the coupling strengths and phases between the external excitation and the two resonances. In the fitting procedure, $\overset{˜}{B_{M}}$ and $\overset{˜}{B_{Q}}$ are taken from the eigenfrequency solver and only allowed to vary within a narrow window (±0.5%), whereas the two coupling coefficient and background *c* are fitted to the calculated transmission spectra. As shown in Figure 5a, the analytical TCMT model successfully reproduces the numerical transmission spectra. This successful fitting indicates that the transmission response of this metasurface is mainly governed by the broad Mie resonance and the narrow qBIC resonance.

**FIGURE 5:**
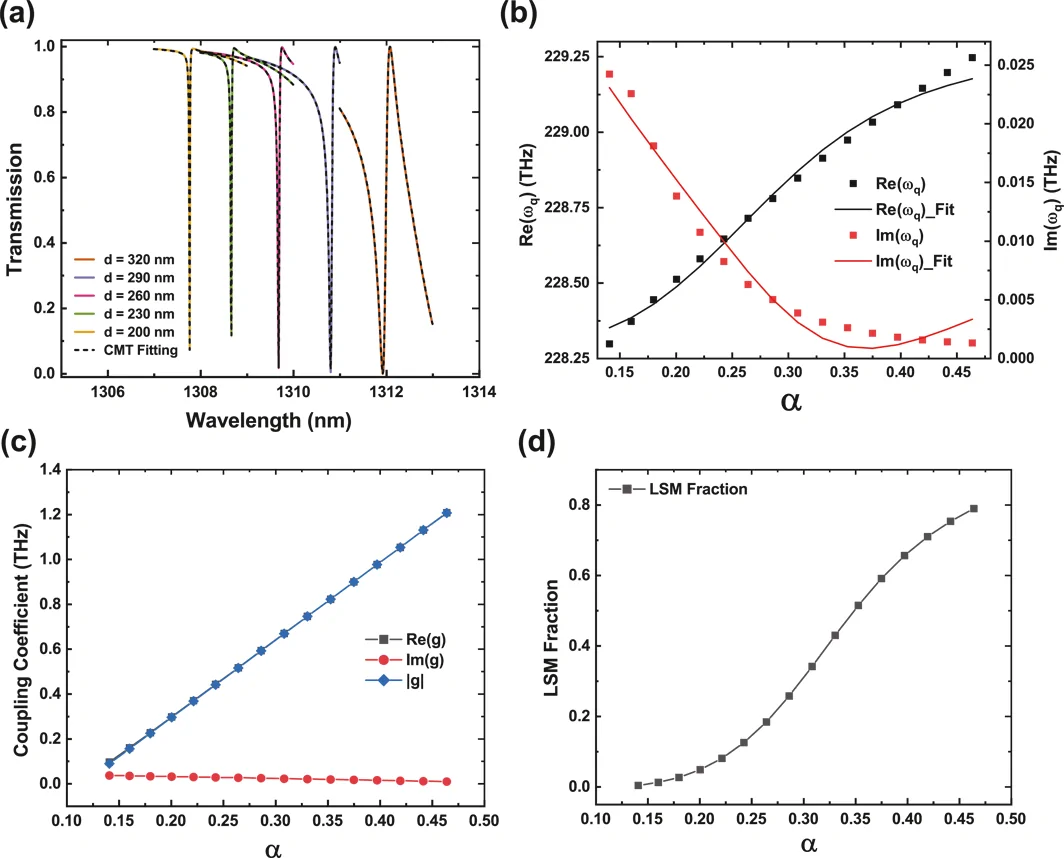
(a) Transmission spectrum under large asymmetry parameters and the fitting results using the proposed TCMT model. (b) Complex eigenfrequency of ${\text{qBIC}}_{1}$ resonance and the fitting results. (c) Variation of the extracted complex coupling strength with the asymmetry parameter α obtained by fitting. (d) Variation of the LSM fraction with the asymmetry parameter α.

However, as discussed above, the Q‐factor of the qBIC resonance increases at large asymmetry parameters due to its coupling with the LSM. Consequently, the complex qBIC frequency obtained from the eigenfrequency solver should not be regarded as the frequency of an isolated bare qBIC. Instead, it can be viewed as a hybridized branch of the coupled qBIC‐LSM system. The LSM is originated from the high‐order diffracted wave propagating along the surface. Away from the Rayleigh anomaly, this diffracted wave behaves mainly as an extended radiative channel. Near the Rayleigh anomaly condition, the in‐plane wave vector becomes close to grazing propagation, and the optical density of states is strongly modified. It can then be approximately described as a discrete mode with a complex frequency. A nonHermitian framework is used to describe the coupling between the qBIC resonance and LSM. The interaction between the qBIC and LSM can therefore be explicitly described by a 2×2 nonHermitian matrix [63, 64]:

(4) $$H\left(\alpha \right)=\left(\begin{array}{cc} \omega_{\alpha }\left(\alpha \right)+i\gamma_{\alpha }\left(\alpha \right) & \tilde{g}\left(\alpha \right)\\ \tilde{g}\left(\alpha \right) & \omega_{\text{LSM}}+i\gamma_{\text{LSM}} \end{array}\right)$$

Here, $\omega_{q}\left(\alpha \right)$ and $\gamma_{q}\left(\alpha \right)$ represents the bare qBIC mode, $\omega_{\mathrm{LSM}}$ and $\gamma_{\mathrm{LSM}}$ describe the LSM. Because the qBIC frequency shifts smoothly with the structural asymmetry parameter α, the bare qBIC dispersion is approximated as $\omega_{q}\left(\alpha \right)=c_{0}+c_{1}\alpha +c_{2}\alpha^{2}$. For a smooth eigenfrequency branch, a quadratic expansion around the center of the fitted parameter range provides the lowest‐order description beyond a purely linear shift, effectively capturing the curvature of the dispersion without introducing excessive free parameters. The loss of bare qBIC mode is modeled as $\gamma_{q}\left(\alpha \right)=\gamma_{0}+A\alpha^{2}$, which is a well‐established result for symmetry‐protected BICs. The coupling coefficient is modeled as a first‐order polynomial to capture its variation trend while avoiding the introduction of too many parameters. Specifically, the coupling coefficient $\tilde{g}\left(\alpha \right)=\left[g_{r0}+g_{r1}\alpha \right]+i\left[g_{i0}+g_{i1}\alpha \right]$ is a complex quantity, where the real part represents the direct coherent coupling, whereas the imaginary part accounts for the dissipative coupling mediated by the shared radiation channels [65, 66]. By diagonalizing this matrix, two hybridized complex‐frequency branches can be obtained.

By fitting these eigenvalues to the numerical results, several physical parameters in H(α) can be extracted, including the effective coupling strength and the mode composition of the hybrid mode. During the fitting procedure, the eigenvalue branch that best matches the qBIC evolution obtained from the full‐wave simulations is identified as the relevant qBIC branch. As illustrated in Figure 5b, both the real and imaginary parts of the extracted complex eigenfrequency closely follow the numerical results over a range of asymmetry parameters. As shown in Figure 5c, as α increases, the real part of coupling strength Re(g) significantly increases, which indicates an enhanced coherent mode coupling, which originates from the increased near‐field spatial overlap between the qBIC resonance and the extended LSM as the asymmetry parameter increases. Conversely, the imaginary part of coupling strength Im(g), remains negligibly small and slightly decreases. This demonstrates that dissipative coupling is strongly suppressed, which implies that the qBIC and the LSM radiate into highly orthogonal far‐field channels. Because they share minimal open radiation channels, their far‐field dissipative interaction remains negligible despite the open nature of the system. To evaluate the impact of this coherent coupling on the system, the corresponding normalized eigenvector for the selected eigenvalue is written as:

(5) $$v=\left[\begin{array}{c} v_{q}\\ v_{\mathrm{LSM}} \end{array}\right]$$

To quantify the mode mixing, the LSM fraction is defined as [67, 68]:

(6) $$\text{LSM}\;\text{fraction}=\frac{|v_{\mathrm{LSM}}{|}^{2}}{{\left|v_{q}\right|}^{2}+|v_{\mathrm{LSM}}{|}^{2}}$$

This metric evaluates the relative contribution of the LSM within the selected hybrid mode. As shown in Figure 5d, when α = 0.14, the LSM fraction is close to zero, indicating that the mode retains a nearly pure qBIC character. However, as α increases, the strong near‐field interaction forces the mode to heavily hybridize with the LSM, causing the LSM fraction to increase, which is consistent with the Q‐factor and field distributions observed in Figure 4. Ultimately, this theoretical framework provides a consistent interpretation. The initial transmission fitting shows that the macroscopic spectral response is governed by the interaction between the Mie resonance and the narrow qBIC resonance. Building on this, the subsequent nonHermitian Hamiltonian analysis further reveals that the observed evolution of the qBIC resonance originates from its hybridization with the LSM.

High‐Q factor is normally preferred for bio‐sensing. As far as we know, the Q factor of qBIC strongly replies on structural asymmetry (Q ∝ $\alpha^{-2}$). A small increase in α will bring dramatic decrease of the Q factor. Conventionally, in order to obtain high Q factor resonance from qBIC, the required asymmetry parameter α is very small. This will make it extremely challenging for nanofabrication. Here, we find that high Q factor could also be obtained with large α and when α≥0.4, the Q factor only changes slightly which will help to alleviate the fabrication difficulties. For example, in bio‐sensing applications, the sensor needs to detect and analyze small variations in the environment, such as slight changes in refractive index and deposition thickness. In most cases, a Q factor of approximately $10^{5}$ is sufficient, which can be achieved with both small (d=420 nm, α=0.0283) and large (d=200 nm, α=0.4638) parameters. The proof‐of‐concept diagram of this sensor is shown in Figure 6a, the silicon block array is submerged in the liqiud analyte with incident light directly from the analyte. A porous plastic substrate with refractive index of 1.33 was used to maintain a homogenous environment [69]. As shown in Figure 6b, for both small and large asymmetry parameters, the ${\text{qBIC}}_{1}$ resonance continuously redshifts as the analyte refractive index increases from 1.328 to 1.332 in steps of 0.002. The bulk sensitivity defined as Δλ/ΔRI is obtained from the linear fitting of both α. As shown in Figure 6c, notably, the sensitivity under the large asymmetry parameter is 605 nm/RIU, which is comparable to the sensitivity of 647.24 nm/RIU observed under small asymmetry parameter. To simulate the effects of biomolecules deposited on the block's surface, as shown in Figure 6d, a dielectric coating with thickness $t_{0}$ (refractive index n = 1.45) was added to the silicon block. As illustrated in Figure 6e,f, the ${\text{qBIC}}_{1}$ resonance continuously redshifts as the thickness of the coating increases from 10 to 40 nm in steps of 10 nm. The surface sensitivity under large asymmetry parameter can achieve 0.095 nm/nm, which is also comparable to the sensitivity of 0.126 nm/nm observed under small asymmetry parameter.

**FIGURE 6:**
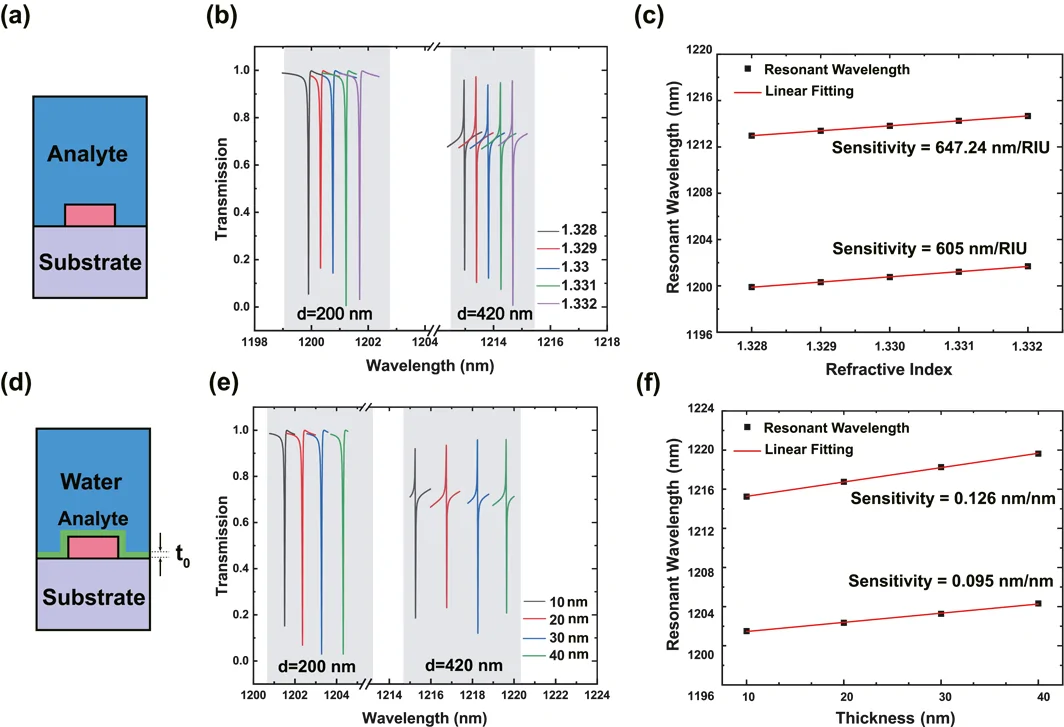
(a) The silicon block array is positioned on a porous plastic substrate with refractive index near 1.33 and immersed in a liquid analyte. (d) The silicon block array is immersed in water (*n* = 1.33), with its surface coated with an analyte layer of thickness $t_{0}$ and refractive index *n* = 1.45. (b), (e) The transmission spectrum ${\text{qBIC}}_{1}$ corresponding to analyte with different refractive index n and the thickness of the analyte $t_{0}$ under small (d = 420 nm, α=0.0283) and large (d = 200 nm, α=0.4638) asymmetry parameter α. (c), (f) Resonant wavelength of ${\text{qBIC}}_{1}$ varies from refractive index n and thickness $t_{0}$ under large and small asymmetry parameters. The sensitivity is calculated by the linear fitting results of both α.

## 4 Conclusion

In conclusion, we propose a silicon square block metasurface array with designed circular notches regarding to the asymmetry parameter α. Two specific BICs supported by the metasurface are indicated as ${\text{qBIC}}_{1}$ and ${\text{qBIC}}_{2}$ and their nonradiative characteristics are explained by point group theory. The evolution of the Q factor of the ${\text{qBIC}}_{1}$ with regard to the α is studied in detail. For small asymmetry (α≤0.14), the Q factor follows the traditional inverse‐square law, governed by the coupling strength between ${\text{qBIC}}_{1}$ and free space. When α>0.14, there is an increase of Q factor. By detailed analysis of coupling strength and EM field distributions, it is found that the coupling between ${\text{qBIC}}_{1}$ and free space is disrupted by the coupling between ${\text{qBIC}}_{1}$ and LSM, where the LSM acts as a nonradiative channel. Stronger coupling between qBIC and LSM will lead to weaker coupling between qBIC and free space, which results in an increase of the Q factor. This discovery not only adds a new theoretical dimension to the design of qBICs but also provides a pathway for the development of more versatile metasurfaces. For refractive index sensing, the bulk sensitivities under both small and large asymmetry parameters can reach 647.24 nm/RIU and 605 nm/RIU, respectively, while maintaining comparable high Q factors ($Q\approx 10^{5}$).

## Author Contributions

**Yi Tian:** conceptualization, investigation, methodology, validation, software, writing – original draft, writing – review and editing, formal analysis. **Qin Fu:** investigation, validation, visualization, writing – review and editing. **Shuai Feng:** writing – review and editing, conceptualization, investigation, methodology. **Qingguo Du:** conceptualization, methodology, investigation, funding acquisition, visualization, project administration, supervision, writing – original draft, writing – review and editing. **Zhengying Li:** validation, visualization, writing – review and editing, project administration. **Fei Xu:** writing – review and editing, visualization, validation.

## Funding

This work was supported by the National Natural Science Foundation of China (Grants Nos: 62375209, 62075173, 62571562 and 12274788).

## Conflicts of Interest

The authors declare no conflicts of interest.

## Supporting information


Supporting Information S1

## Data Availability

The data that support the findings of this study are available from the corresponding author upon reasonable request.
