# Supplementary material for: Increasing of Q Factor of Quasi‐Bound States in the Continuum With Increased Asymmetry Parameter in a Silicon Metasurface Array
Source: Nanophotonics. 2026 Jul 15;15(14):e70208. doi: 10.1002/nap2.70208 (PMC13642079; doi:10.1002/nap2.70208)
Supplement: Supplementary file 1 [file NAP2-15-e70208-s001.pdf]

Supplementary Information for

Increasing of Q factor of Quasi-Bound States in the Continuum with Increased Asymmetry Parameter in a Silicon Metasurface Array

Yi Tian[1], Qin Fu[1, *], Shuai Feng[2, †], Qingguo Du[1,‡], Zhengying Li[1,3] and Fei Xu[4,5]

[1] School of Information Engineering, Wuhan University of Technology, Wuhan 430070, China

[2] School of Science, Minzu University of China, Beijing 100081, China

[3] National Engineering Research Center of Optical Fiber Sensing Technology and Networks, Wuhan University of Technology, Wuhan 430070, China

[4] National Laboratory of Solid State Microstructures, Nanjing University, Nanjing 210093, China

[5] College of Engineering and Applied Sciences, Nanjing University, Nanjing 210093, China

* fuqin@whut.edu.cn

† shuaifeng@muc.edu.cn

‡ qingguo.du@whut.edu.cn

This file includes:

S1: Results of the mesh convergence test

S2: The symmetry characteristics of $Mode_1$ and $Mode_4$

S3: The formula for the multipole decomposition of $qBIC_1$

S4: The effects of small variations in asymmetry parameter on the qBIC resonance

S5: The mismatch of Q factor and the coupling strength

S6: The formula to calculate the wavelength of LSM

S7: Q-factor enhancement of the $qBIC_2$ resonance

S8: Parameter sweep results for silicon block thickness t and period P

S9: Another case with a non–center-symmetric notch

S1. Results of the mesh convergence test

To further verify the reliability of our simulation, a mesh convergence analysis was performed under a large asymmetry factor (d = 220 nm), where sharp corners are introduced in the geometry.

As illustrated in Fig. S1(a), the transmission spectra calculated with minimum mesh sizes of 1.5 nm, 2 nm, and 3 nm are identical. However, a slight spectral shift (~0.01 nm) occurs when the minimum mesh size is increased to 5 nm. This suggests that the simulation results are fully converged when the minimum mesh size is 3 nm or smaller. Additionally, to evaluate the influence of sharp corners, a comparison was made between the original structure with sharp edges and a modified structure where the sharp corners were replaced with a rounded corner with a radius of 1.5 nm. As shown in Fig. S1 (b), negligible differences are observed in the transmission spectra of the two cases. This demonstrates that the presence of sharp corners does not significantly affect the optical response of the structure.

These results confirm that the adopted mesh settings provide reliable simulation accuracy.

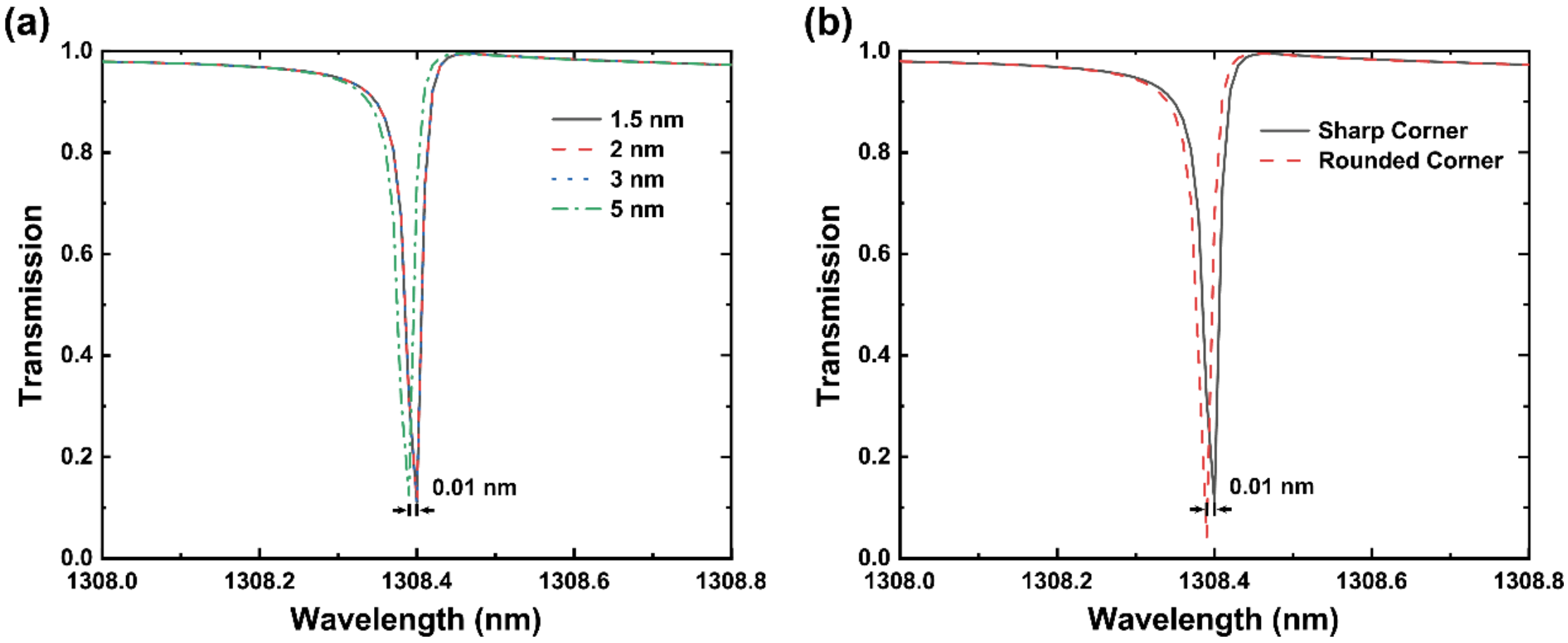

Fig. S1. (a) Transmission spectra (d = 220 nm) calculated using different minimum mesh sizes for the silicon block (1.5, 2, 3, and 5 nm). (b) Transmission spectra calculated with a minimum mesh size of 1.5 nm with and without sharp corners, respectively.

S2. The symmetry characteristics of $Mode_1$ and $Mode_4$

A C_{4v} lattice exhibits eight symmetry operations, indicated as $\{E, C_4, C_4^{-1}, C_2, \sigma_x, \sigma_y, \sigma_d^{'}, \sigma_d^{''}\}$. The identity operation (E) leaves all points unchanged, representing no transformation. The C_4 rotation corresponds to a 90° counterclockwise rotation about the z-axis, while the C_4^{-1} operation represents a 90° clockwise rotation. A C_2 rotation, equivalent to a 180° rotation about the z-axis. The group also includes four reflection operations. The σ_x reflection occurs in the xz-plane and changes (x, y, z) into (x, −y, z), while the σ_y reflection in the yz-plane gives (x, y, z) → (−x, y, z). The two diagonal reflections, $\sigma_d^{'}$ and $\sigma_d^{''}$, correspond to reflections through the planes x = y and x = −y, resulting in transformations (x, y, z) → (y, x, z) and (x, y, z) → (−y, −x, z), respectively. These eight symmetry operations are represented by black arrows on the eigenfield profile (H_z) shown in Fig. S1. The character table of the C_{4v} point group is shown in Table S1.

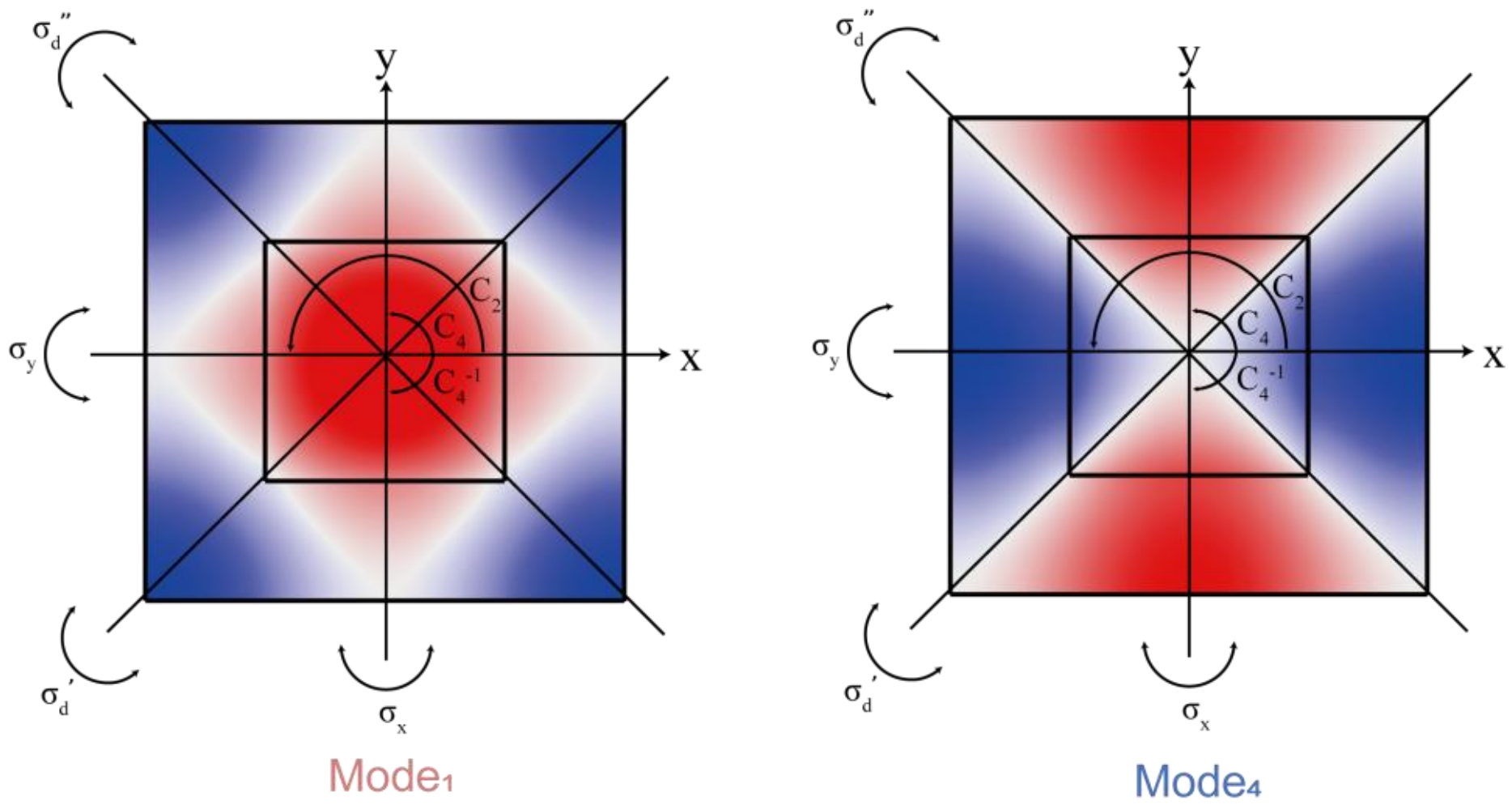

Fig. S1. The mode profiles of H_z for mode$_1$ and mode$_4$ with the symmetry operations of C_{4v} group indicated by arrows.

Table S1. Character table of the C_{4v} point group

C_{4v}	E	$2C_4$	C_2	σ_x, σ_y	$\sigma_d^{'}, \sigma_d^{''}$
A_1	1	1	1	1	1
A_2	1	1	1	-1	-1
B_1	1	-1	1	1	-1
B_2	1	-1	1	-1	1
E	2	0	-2	0	0

S3. The formulas for the multipole decomposition of qBIC$_1$

The multipole decomposition in Cartesian coordinates are calculated respectively with following formulas (higher order dipoles are neglected during the calculation owing to the extremely weak influence on the scattering powers)

The moments of electric dipole (ED) **p** and magnetic dipole (MD) **m** are expressed as

$$\mathbf{p} = \int_V \left[\mathbf{P}(\mathbf{r}) j_0(kr) + \frac{k^2}{10} \left[(\mathbf{r} \cdot \mathbf{P}(\mathbf{r}))\mathbf{r} - \frac{1}{3} r^2 \mathbf{P}(\mathbf{r}) \right] \frac{15 j_2(kr)}{(kr)^2} \right] d^3 r$$

$$\mathbf{m} = -\frac{i\omega}{2} \int_V [\mathbf{r} \times \mathbf{P}(\mathbf{r})] \frac{3 j_1(kr)}{kr} d^3 r$$

where $\mathbf{P(r)} = \varepsilon_0(\varepsilon_r - n_b^2)\boldsymbol{E}(\boldsymbol{r})$ represents the induced polarization vector at position $\boldsymbol{r}$ inside the silicon block, n_b represents the refractive index of the surrounding background medium, and ε_r is the relative permittivity of the silicon block. $j_n(kr)$represents the spherical Bessel function of the first kind of order n (n = 0, 1, 2, 3). k represents the wavenumber in the surrounding medium and $r = |\boldsymbol{r}|$ represent its radial distance.

The moments of electric quadrupole (EQ) $\boldsymbol{Q}_{\alpha\beta}$ and magnetic quadrupole (MQ) $\boldsymbol{M}_{\alpha\beta}$ are expressed as:

$$
\boldsymbol{Q}_{\alpha\beta} = 3\int_V \left\{ \left[r_\alpha P_\beta + r_\beta P_\alpha - \frac{2}{3}(\mathbf{r}\cdot\mathbf{P}(\mathbf{r}))\delta_{\alpha\beta} \right] \frac{j_1(kr)}{kr} + 2k^2 \left[5 r_\alpha r_\beta (\mathbf{r}\cdot\mathbf{P}(\mathbf{r})) - r^2 (r_\alpha P_\beta + r_\beta P_\alpha) - r^2 (\mathbf{r}\cdot\mathbf{P}(\mathbf{r}))\delta_{\alpha\beta} \right] \frac{j_3(kr)}{(kr)^3} \right\} d^3 r
$$

$$
\boldsymbol{M}_{\alpha\beta} = \frac{\omega}{3i}\int_V \left[r_\alpha (\mathbf{r}\times\mathbf{P}(\mathbf{r}))_\beta + r_\beta (\mathbf{r}\times\mathbf{P}(\mathbf{r}))_\alpha \right] \frac{15 j_2(kr)}{(kr)^2} d^3 r
$$

where $\alpha, \beta \in \{x, y, z\}$, r_α and P_α represent the specific Cartesian components of the position vector $\boldsymbol{r}$ and the polarization vector $\mathbf{P(r)}$, respectively. Using the multipole moments, it is easy to obtain the total scattering cross section as

$$
C_{\text{sca}}^{\text{ED}} = \frac{k^4}{6\pi\varepsilon_0^2 n_b^4 E_0^2} |\mathbf{p}|^2
$$

$$
C_{\text{sca}}^{\text{MD}} = \frac{k^4 \mu_0}{6\pi\varepsilon_0 n_b^2 E_0^2} |\mathbf{m}|^2
$$

$$
C_{\text{sca}}^{\text{EQ}} = \frac{k^6}{720\pi\varepsilon_0^2 n_b^4 E_0^2} \sum_{\alpha,\beta} \left| \boldsymbol{Q}_{\alpha\beta} \right|^2
$$

$$
C_{\text{sca}}^{\text{MQ}} = \frac{k^6 \mu_0}{720\pi\varepsilon_0 n_b^2 E_0^2} \sum_{\alpha,\beta} \left| \boldsymbol{M}_{\alpha\beta} \right|^2
$$

where E_0 represents the electric field amplitude of the incident plane wave.

S4: The effects of small variations in asymmetry parameter on the qBIC resonance

As shown in Fig. S2(a), a small variation in d causes a shift of 0.282 nm in the resonance wavelength under large α, which is smaller than the shift of 0.479 nm observed under small α. As shown in Fig. S2(b) and (c), linear fitting is applied to evaluate the rate of change in the Q factor with respect to variations in α. Under small α, the rate of change is 2.54×10^7, which is two orders of magnitude greater than that under large α (1.9×10^5). This demonstrates that small variations in the asymmetry parameter have a much greater influence on the Q factor under small-asymmetry conditions than under large-asymmetry conditions.

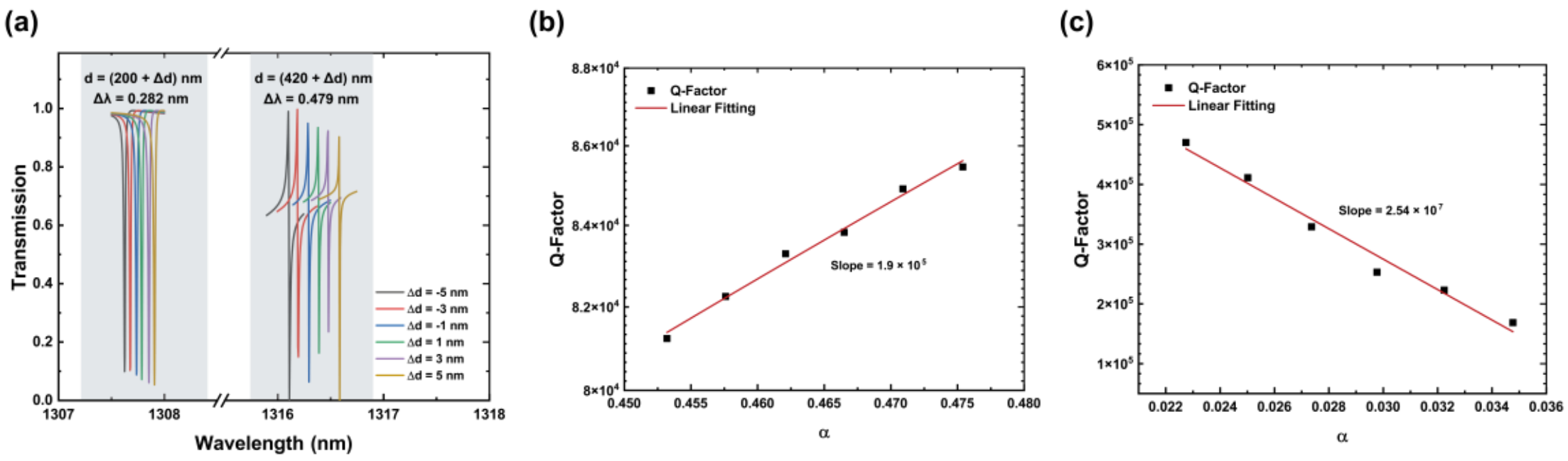

Fig. S2. (a) Spectra of the $qBIC_1$ resonance under large and small asymmetry parameters when the distance between the center of the circle and the silicon block varies from -5 to 5 nm. (b)(c) Q factor of the $qBIC_1$ resonance as a function of small variations in the asymmetry parameter α under large and small asymmetry parameters, with the linear fitting results shown by the red curve, respectively.

S5. The mismatch of Q factor and the coupling strength

When α=0.1407, the Q factor does not match the coupling strength. This slight mismatch indicates that the Mie resonance slightly influences the radiative properties of qBIC. For verification purposes, the Q factor of the $qBIC_1$ is calculated in the eigenfrequency solver, which excludes the influence of the Mie resonance. As shown in Fig. S3, the increase in Q_{eigen} perfectly matches the decrease in coupling strength shown in Fig. 4(b) with rising asymmetry parameter α.

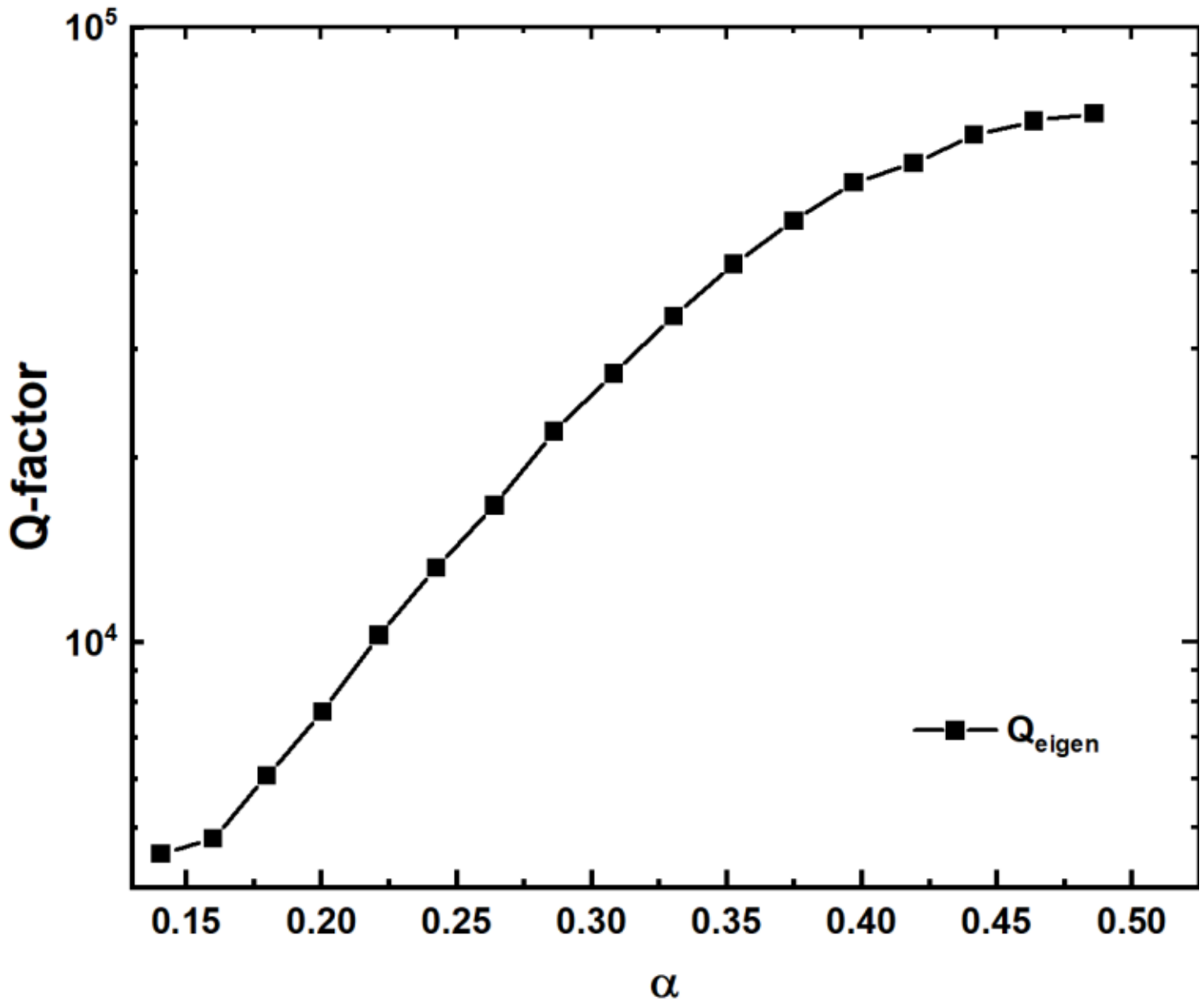

FIG. S3. The Q factor calculated in eigenmode solver vary with symmetry parameter α.

S6. The formula to calculate the wavelength of LSM

The Lattice Surface Mode (LSM) originates from the higher-order diffraction of the incident light wave. When the wavevector of the diffracted light matches the wavevector provided by the periodic structure, it transforms into an evanescent wave propagating along the surface, which is named as LSM. The dispersion relation for a square lattice is governed by:

$$k_0^2 = [k_x + m(2\pi / P)]^2 + [k_y + n(2\pi / P)]^2$$

where $k_0 = 2\pi n / \lambda$, $k_x = ((2\pi n) / \lambda)\sin\theta$ and $k_y = ((2\pi n) / \lambda)\sin\varphi$, k_0 is the wave vectors of the diffracted light, k_x and k_y represents the wave vectors of incident light in x and y directions, respectively. n is the refractive index of the surrounding medium, and (m,n) are integers defining the diffraction orders in the x and y directions, respectively. If the incident angles along x and y directions are varied independently, the wavelength of the LSM can be expressed as

$$\lambda_{(\pm1,0)} = nP(1 \pm \sin\theta)$$
$$\lambda_{(0,\pm1)} = nP(1 \pm \sin\varphi)$$

When light incident normally, the $(0, \pm1)$ and $(\pm1,0)$ diffraction orders are degenerate. For the proposed metasurface with a period P = 900 nm and a refractive index n = 1.45, the wavelength of LSM can be calculated by $\lambda_{LSM} = n \times P = 1305nm$.

S7. Q-factor enhancement of the qBIC$_2$ resonance

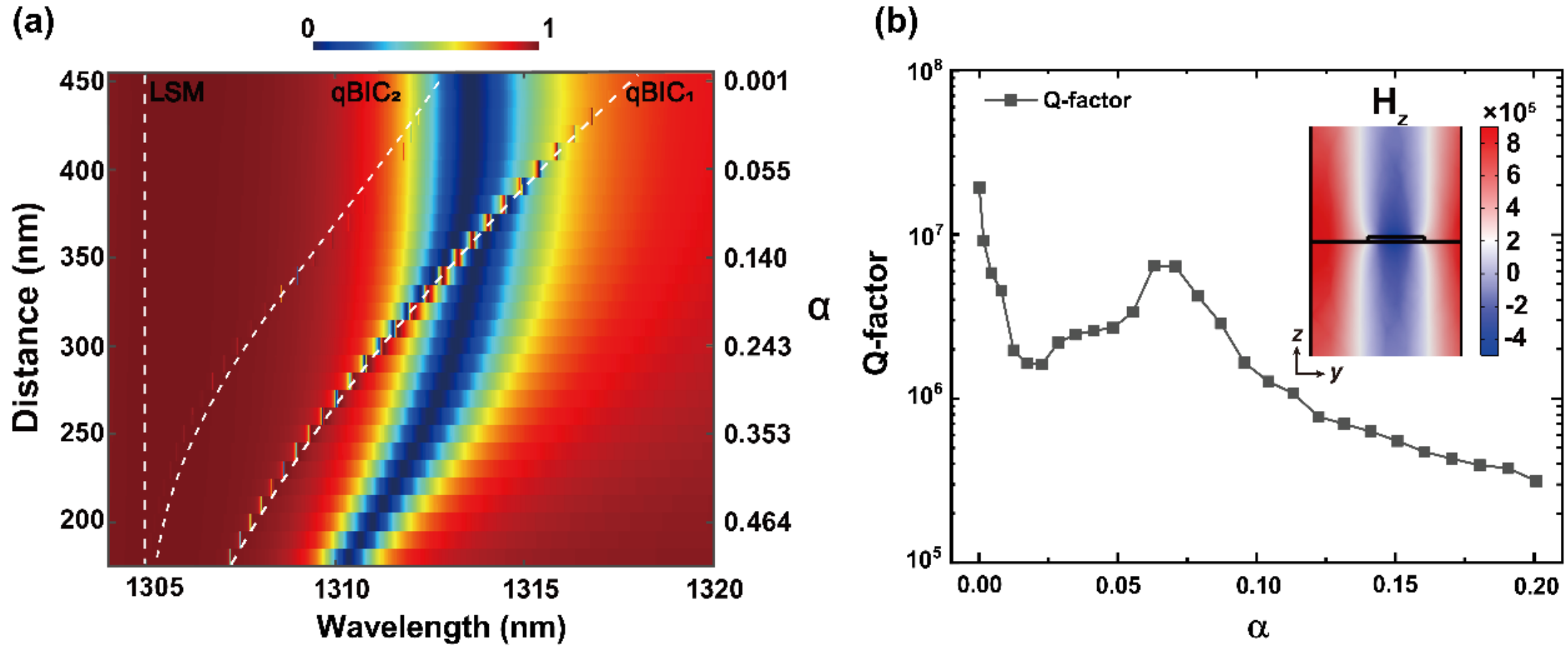

FIG. S4. (a) The transmission spectrum varies of *d*. The color indicates transmittance, with the colorbar above the image. The wavelength of LSM, qBIC$_1$ and qBIC$_2$ are highlighted in the figure using white dashed lines. (b)The Q-factor of qBIC$_2$ resonance varied the asymmetry parameter α.

The underlying mechanism of qBIC$_2$ is identical to qBIC$_1$. As shown in Fig. S4(a), when the asymmetry parameter increases, both the qBIC$_1$ and qBIC$_2$ resonances blue shift, approaching the wavelength of the LSM. Their linewidths also exhibit a trend of initially increasing and then decreasing. The evolution of the Q factor of qBIC$_2$ is presented in Fig. S4(b). Similar to qBIC$_1$, the Q-factor of qBIC$_2$ initially decreases with increasing asymmetry, followed by a an increase as the resonance approaches the LSM. When the qBIC$_2$ resonance becomes excessively close to the LSM, the Q-factor decreases again. The inset of Fig. S4(b) shows the H_z field distribution in the y–z plane at $\alpha =$ 0.055. A clear magnetic field directed along the -z direction appears at the center of the Si blocks, while the magnetic fields on both sides point in the +z direction. This field distribution indicates that the mode propagates along the surface in the y direction, confirming the involvement of LSM. This observation aligns with the field characteristics observed for qBIC$_1$ in Fig. 4, as presented in our response to Comment 1 of Reviewer 2.

Further evidence can be found in the extinction spectra shown in Figure. 4(b). As the asymmetry parameter increases, distinct extinction peaks gradually emerge for both qBIC$_1$ and qBIC$_2$. This clearly demonstrates that the coupling between the qBIC resonances and the LSM directs more energy along the surface, thereby reducing radiation into free space. As a result, a larger fraction of the resonance energy is confined within the surface-propagating mode, leading to the enhancement of the Q factor. The similar evolution of qBIC$_1$ and qBIC$_2$ in terms of linewidth, extinction behavior, and field distribution indicates that both the two resonances share the same coupling mechanism with the LSM.

S8. Parameter sweep results for silicon block thickness t and period P

As illustrated in Fig. S5 (a)–(e), when the thickness of the silicon block t is varied from 25 nm to 45 nm in 5 nm steps, both qBIC$_1$ and Mie resonances exhibit a continuous redshift. Fig. S6(f) illustrates the resonance wavelengths of qBIC$_1$, Mie, and LSM as a function of thickness t. The

position of LSM remains unchanged with thickness, causing the $qBIC_1$ resonance to shift further from LSM as the thickness increases. This will reduce the growth rate of the Q-factor. As shown in Fig. S6(a)–(e), while the inflection point remains nearly unchanged, the maximum Q-factor at the largest asymmetry parameter (α = 0.5) decreases from 1×10^5 to 2×10^4 as the thickness increases from 30 nm to 45 nm. This phenomenon indicates that the growth rate of Q-factor can be controlled by the thickness of the Si block, while the inflection point remains stable.

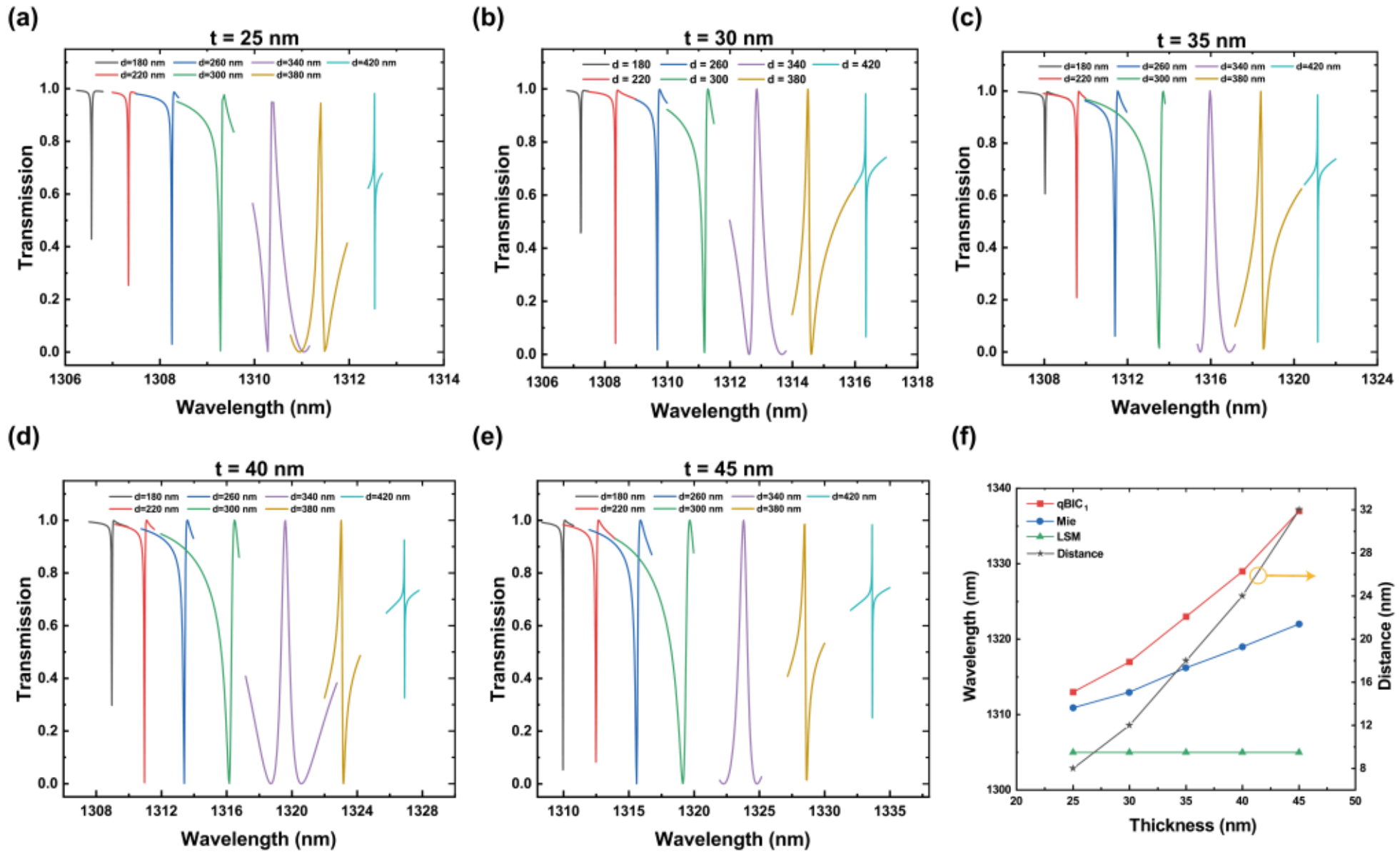

FIG. S5 (a)-(e) The transmission spectrum of the metasurface at different values of d as t varies from 25 to 45 nm in steps of 5 nm. (f) The resonance wavelengths of $qBIC_1$, Mie, and LSM as a function of the thickness t are represented by red, blue, and green lines, respectively, while the distance between $qBIC_1$ and LSM is indicated by a black line.

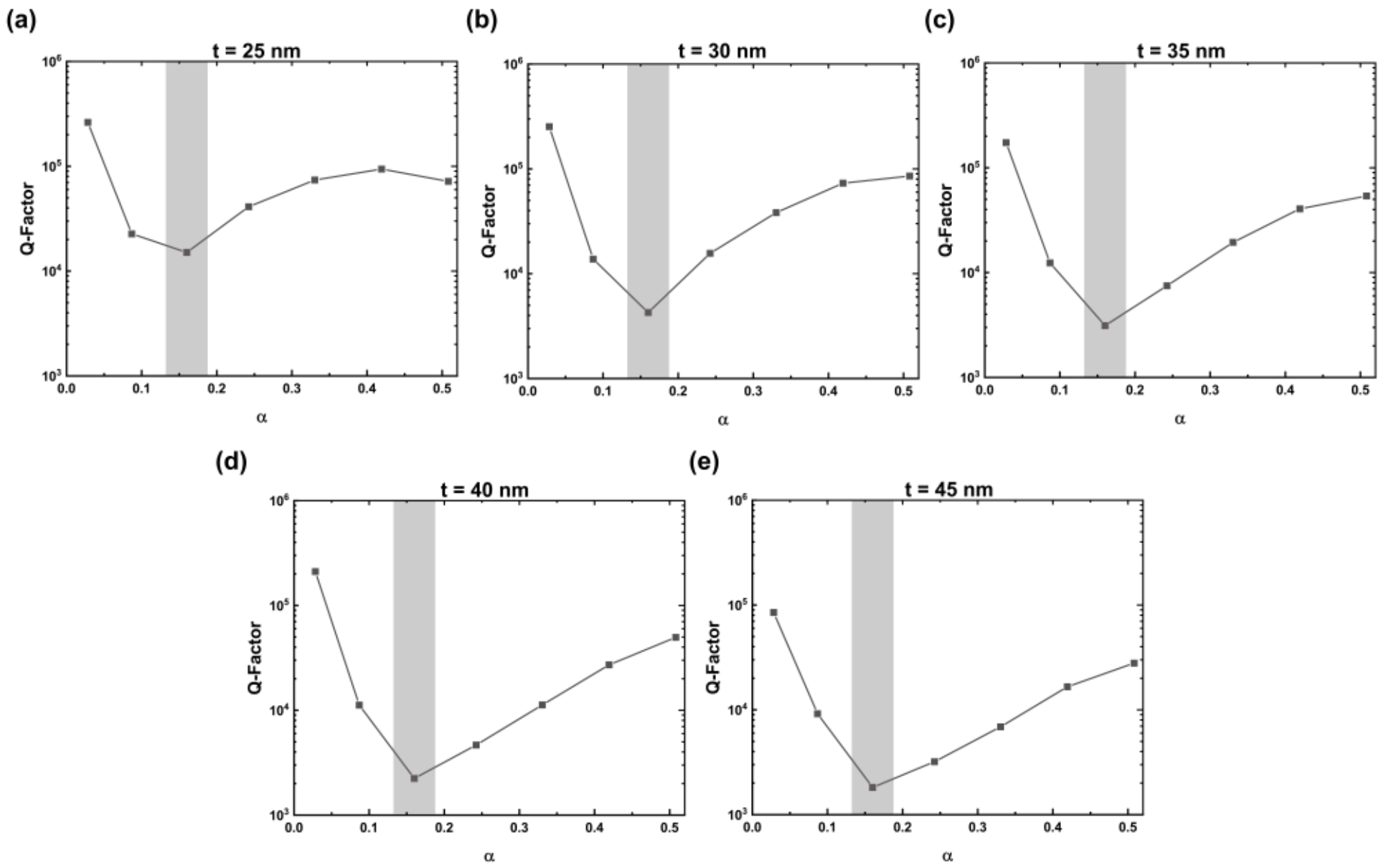

FIG. S6 (a)-(e) The Q-Factor of $qBIC_1$ at different values of α as t varies from 25 to 45 nm in steps of 5 nm with inflection point marked by gray shading.

As shown in Fig. S7(a)–(e), when the period P varies from 800 nm to 1000 nm in steps of 50

nm, both the $qBIC_1$ and TD resonances exhibit a continuous redshift, but $qBIC_1$ shifts more slowly than TD and LSM. This causes the position of the $qBIC_1$ resonance to move from the right side of the TD resonance to the left, approaching the LSM. As shown in Fig. S8(a)–(e), the inflection point of the Q factor of $qBIC_1$ resonance versus the asymmetry factor decreases as *P* increases. However, if $qBIC_1$ becomes too close to LSM, the modulation depth decreases and the resonance eventually vanishes.

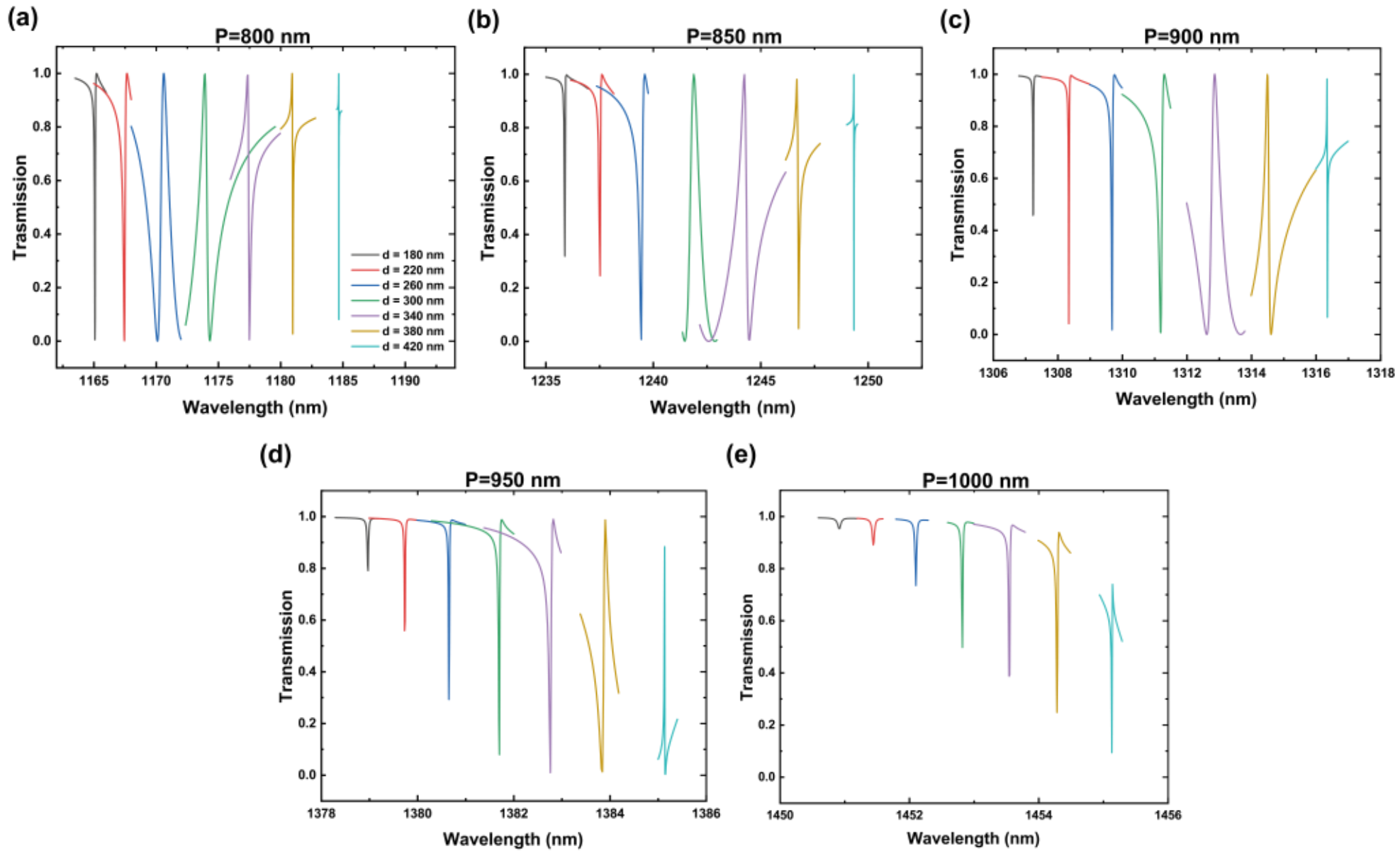

Fig. S7. (a)-(e) The transmission spectra of the metasurface at different values of d as P varies from 800 to 1000 nm in steps of 50 nm.

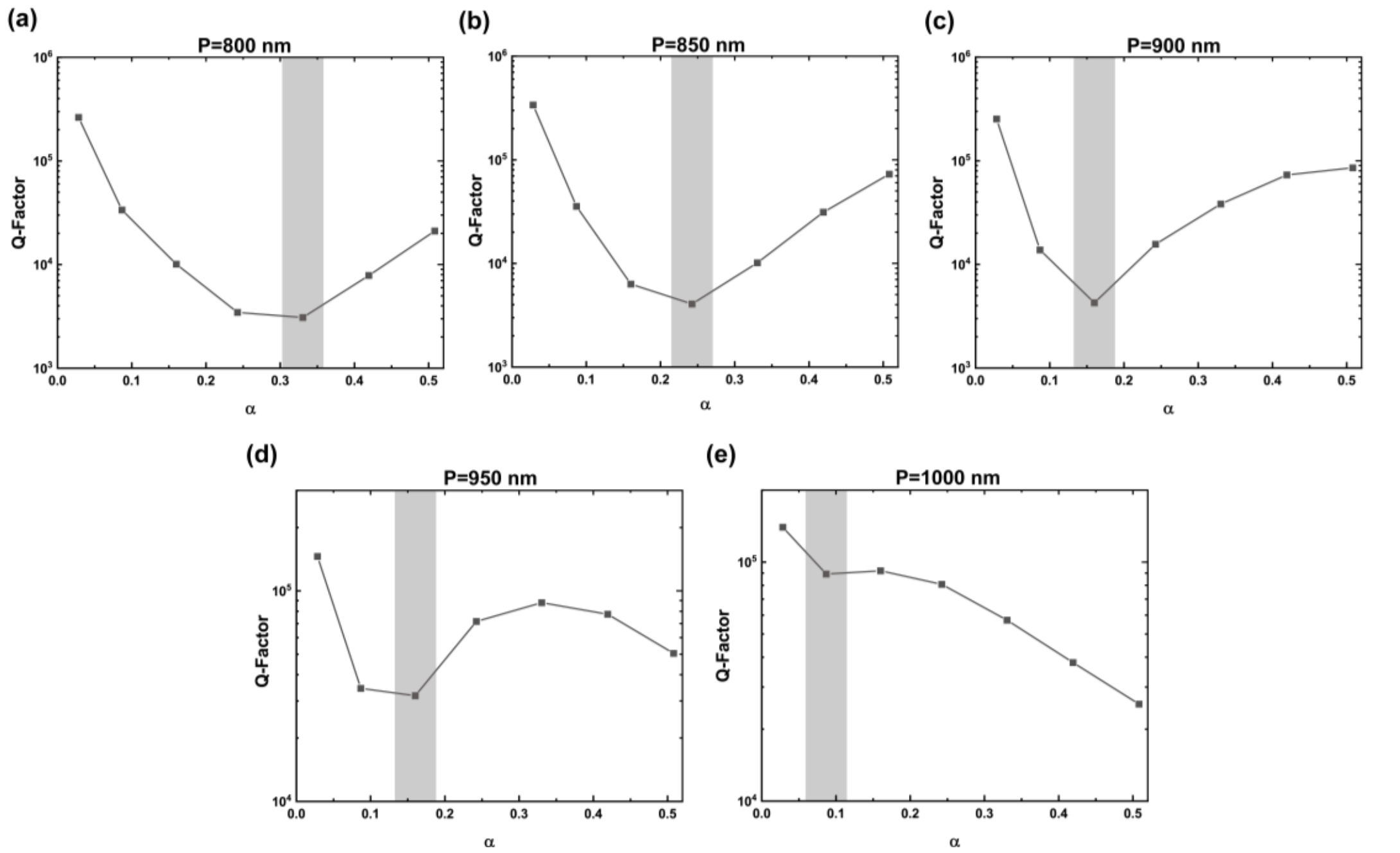

Fig. S8. (a)-(e) The Q-Factor of $qBIC_1$ at different values of α as P varies from 800 to 1000 nm in steps of 50 nm with inflection point marked by gray shading.

S9. Another case with a non–center-symmetric notch

Additional simulations are performed in which the center of the circular notch is displaced by Δ, away from the axis of symmetry. As shown in Fig. S9(a), where the asymmetry parameter is fixed at 0.055, but the center of the circular notch is displaced by a distance Δ from the axis of symmetry. When Δ varies from 10 nm to 60 nm, the Q-factor of the $qBIC_1$ resonance decreases from 41,528 to 32,955. As shown in Fig. S9(b), however, the Q-factor of the $qBIC_1$ resonance first decreases and then increases with the increase of the asymmetry parameter α. This trend does not change as displacement Δ increases, indicating that for a fixed perturbation path (as investigated in this study), the dependence of the Q-factor on the asymmetry parameter remains governed by the symmetry-breaking mechanism.

The results confirm that although the resonance characteristics are modified, the overall trend of Q factor evolution with respect to the asymmetry parameter remains consistent.

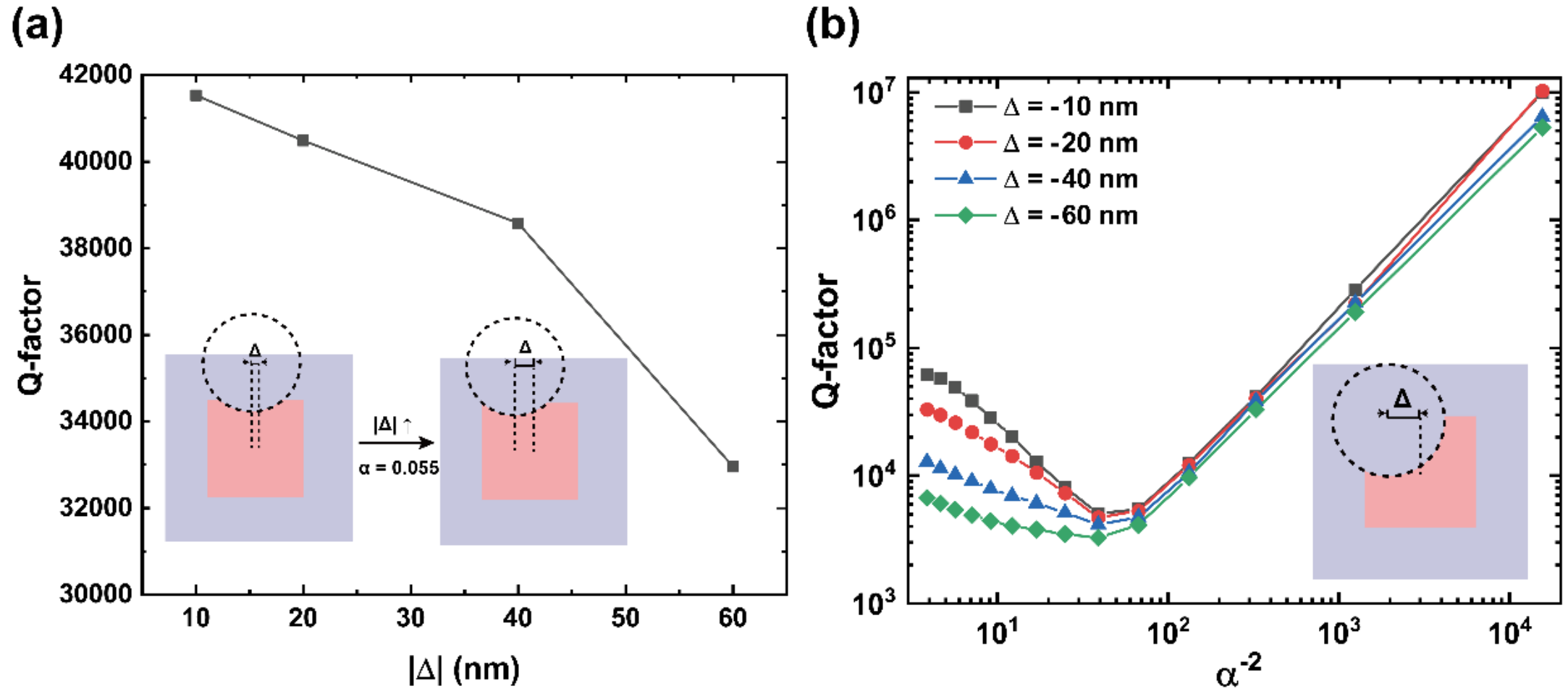

Fig. S9. (a) Variation of the Q factor of the $qBIC_1$ resonance as a function of the center displacements Δ, with the asymmetry parameter fixed at 0.055. (b) Transmission spectra of y-polarized light under x-polarized incidence at different values of *d* when Δ = 60 nm.
